# Regulation of MAIT cells through host-derived antigens

**DOI:** 10.3389/fimmu.2024.1424987

**Published:** 2024-06-24

**Authors:** Emi Ito, Sho Yamasaki

**Affiliations:** ^1^ Department of Molecular Immunology, Research Institute for Microbial Diseases (RIMD), Osaka University, Suita, Japan; ^2^ Laboratory of Molecular Immunology, Immunology Frontier Research Center (IFReC), Osaka University, Suita, Japan; ^3^ Center for Infectious Disease Education and Research (CiDER), Osaka University, Suita, Japan

**Keywords:** MAIT cell, MR1 ligand, bile acids, T cell development, self-antigen

## Abstract

Mucosal-associated invariant T (MAIT) cells are a major subset of innate-like T cells that function at the interface between innate and acquired immunity. MAIT cells recognize vitamin B2-related metabolites produced by microbes, through semi-invariant T cell receptor (TCR) and contribute to protective immunity. These foreign-derived antigens are presented by a monomorphic antigen presenting molecule, MHC class I-related molecule 1 (MR1). MR1 contains a malleable ligand-binding pocket, allowing for the recognition of compounds with various structures. However, interactions between MR1 and self-derived antigens are not fully understood. Recently, bile acid metabolites were identified as host-derived ligands for MAIT cells. In this review, we will highlight recent findings regarding the recognition of self-antigens by MAIT cells.

## 1 Introduction

Mucosal-associated invariant T (MAIT) cells are the most abundant T cell subset in humans (1). They recognize non-peptidic antigens presented on a monomorphic antigen presenting molecule, MR1 (2–4). T cell receptor (TCR) repertoires of MAIT cells are composed of restricted TCRα and β chains (mice, TRAV1- TRAJ9/12/33–TRBV13/19; human, TRAV1-2- TRAJ12/20/33–TRBV6/20) that recognize riboflavin-based metabolites produced in microbes but not in mammals (2, 5, 6).

MAIT cells are positively selected in the thymus through the interaction with MR1-expressing double-positive (DP) thymocytes (7) and/or thymic epithelial cells (8). The development of MAIT cells is severely impaired in germ-free (GF) mice and microbiota-derived antigen 5-OP-RU is reported to contribute to their thymic selection (8, 9). Judging from its structure, 5-OP-RU is unlikely to cross the plasma membrane and, as no transporter proteins have been identified thus far, it is still unclear how unstable 5-OP-RU is transferred from gut to the thymus. Additionally, a small but significant number of MAIT cells have been detected in the thymi of GF mice (5, 8, 10), potentially suggesting the presence of endogenous antigen(s) that influence thymic development of MAIT cells (11).

Conventional T cells, which recognize peptidic antigens presented by classical MHC molecules using variant TCRs, are positively selected by weak affinity of self-peptides (12). In the periphery, self-peptides also contribute to the survival and maintenance of conventional T cells (13–15). Since the affinity of bacteria-derived 5-OP-RU to MAIT TCR is strong enough to induce negative selection (8), it is possible that host-derived weak antigen(s) may also be involved in the development and/or maintenance of MAIT cells. However, it is still unclear whether the strength of antigen affinity can differentially regulate the fate and function of MAIT cells, as is reported for conventional T cells.

One of the tissues in which human MAIT cells are most abundant is the liver, particularly in the hepatic sinusoid around bile ducts (16–19). Liver MAIT cells constitutively express activation markers, suggesting that MAIT cells receive continuous TCR signaling even in a steady state (20, 21). Thus, the unique localization and/or maintenance of tissue residency may also be regulated by tissue-derived endogenous factor(s) abundant in the liver. However, such self-antigens have not been identified thus far.

## 2 Diverse ligands presented by MR1

### 2.1 Diversity and specificity of MR1 ligands

MR1 is a well-conserved MHC class I-like molecule and present small compounds unlike CD1 molecules which can accommodate large lipids. For ligand binding, MR1 utilizes the A-pocket, which is flexible and accommodates a large variety of ligands (22, 23). Within the A-pocket, K43 mediates a covalent bond with some typical antigens (5-OP-RU, 6-FP and Ac-6-FP) (24, 25). Neutralization of this positively charged K43 is required for the stabilization of MR1 (22). However, some other ligands (RL-7-Me, RL-6, 7-diMe, diclofenac (DCF), DB28 and NV18.1) non-covalently bind to MR1 (2, 22, 23, 26, 27). It is unknown whether these ‘non-covalent’ ligands induce MR1 stabilization beyond K43 neutralization. MR1 additionally requires the ligand to possess a hydroxy group to be ‘pinched’ by two Arg residues found on MR1 (R9 and R94) (2, 6, 22). However, a comprehensive screening of potential MR1 ligands demonstrates that MR1 can actually present a notably broader range of small molecules regardless of these requirements, including mono- and multi-cyclic chemical compounds (22, 23). It is therefore possible that MR1 can bind to previously unappreciated endogenous metabolites.

### 2.2 Self recognition by MAIT cells

There are some studies that support the possibility of self-recognition by MAIT cells. Young et al. reports that a cell line expressing a MAIT TCR was activated in the presence of MR1-expressing antigen presenting cells (APC) in the absence of infection (28). Additionally, cancer cells have been shown to be targeted by MAIT cells utilizing an MR1-dependent mechanism, although with an unidentified ligand (29). Some atypical MR1-related T cells (MR1T cells) are reported to respond to self-derived antigen(s) (30, 31). Recently, Chancellor et al. discovered the rare occurrence of self-reactive MAIT cells that display unique T-helper functions (32). However, endogenous MAIT cell antigen(s) presented by MR1 are yet to be identified.

## 3 Bile acid metabolites as host-derived ligands

### 3.1 Cholic acid 7-sulfate is a MAIT cell ligand

We recently purified and identified cholic acid 7-sulfate (CA7S) as a host-derived ligand for MAIT cells (33). CA7S has a structure that is distinct from those of previously-reported MR1 ligands in that it uniquely possesses four rings. In addition to previously-demonstrated small ligands (2, 6, 22), a large cholane skeleton can also be accommodated within the MR1 pocket. Notably, a competition assay (34) revealed that CA7S binds to the A-pocket of the MR1 molecule (33) similar to known ligands (2, 25). However, the chemical structure of CA7S and its ability to increase surface expression of MR1 lacking K43 (K43A) suggested that CA7S could bind to MR1 without forming a Schiff base. Indeed, the relative affinity of CA7S to MR1 was estimated to be as weak as that of DCF (33), which is also a non-covalent ligand (22). One of the structural characteristics of CA7S is the presence of carboxy group at position 24. This moiety might be interacted with cationic residues in the A-pocket, which warrants further structural analysis. Although we reported a potential function of CA7S on MAIT cell development, comprehensive understanding of its role in MAIT cell biology need further investigation.

### 3.2 Role of CAS in bile acid metabolic pathway

**Figure 1:**
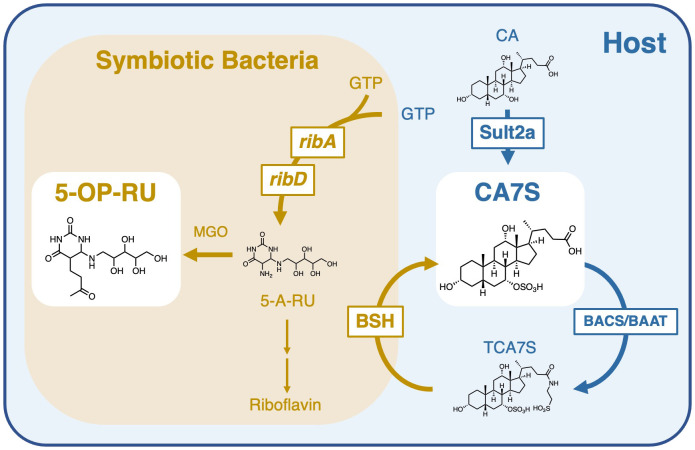
Role of symbiotic bacteria in the generation and modification of MAIT cell antigens. Bacterial riboflavin biosynthesizing enzymes, such as *ribA* and *ribD*, generate 5-A-RU, which is converted to 5-OP-RU in the presence of methylglyoxal (MGO) (left). CAS is produced by sulfation of cholic acid (CA) by sulfotransferase 2a (Sult2a) in the host. CAS is further taurine-conjugated in the host by bile acid–CoA:amino acid N-acyltransferase (BAAT) or bile acid–CoA synthetase (BACS) to generate TCA7S. Most intestinal bacteria have deconjugation enzymes, bile salt hydrolases (BSH), which metabolize TCA7S to CA7S. Symbiotic bacteria are therefore required for the maintenance of both 5-OP-RU and CA7S.

CA7S is a primary bile acid produced from cholic acid by sulfotransferase 2a (Sult2a), which is bile acid-specific sulfotransferases (35–37) (**Figure 1**). As amphiphilic bile acids are sometimes toxic, sulfotransferase function is essential for neutralization and detoxification of bile acids like cholic acid. To do this, Sult2a adds a hydrophilic SO_3_^−^ group to the hydrophobic cholane skeleton, which generates sulfated cholic acids for excretion in feces and urine (35, 38). Thus, the significance of CA7S was previously thought to be as an excreted bile acid metabolite, and only a few additional roles were reported (39, 40).

Although CA7S is biosynthesized in the host, levels of CA7S were decreased in GF mice (33). This is likely due to the lack of deconjugation of tauro-CA7S (TCA7S) by symbiotic bacteria (**Figure 1**), as TCA7S was increased in GF mice (33, 41). Thus, CA7S is an endogenous metabolite, but its quantities are largely influenced by symbiotic bacteria. This is consistent with the observation that MAIT cells dramatically decreased in GF mice (5, 8, 9). These results suggest that the reduction in CA7S levels might also contribute to the impairment of MAIT cell development in GF mice in combination with the lack of microbial antigens like 5-OP-RU.

### 3.3 Role of CAS in MAIT cell development

**Figure 2:**
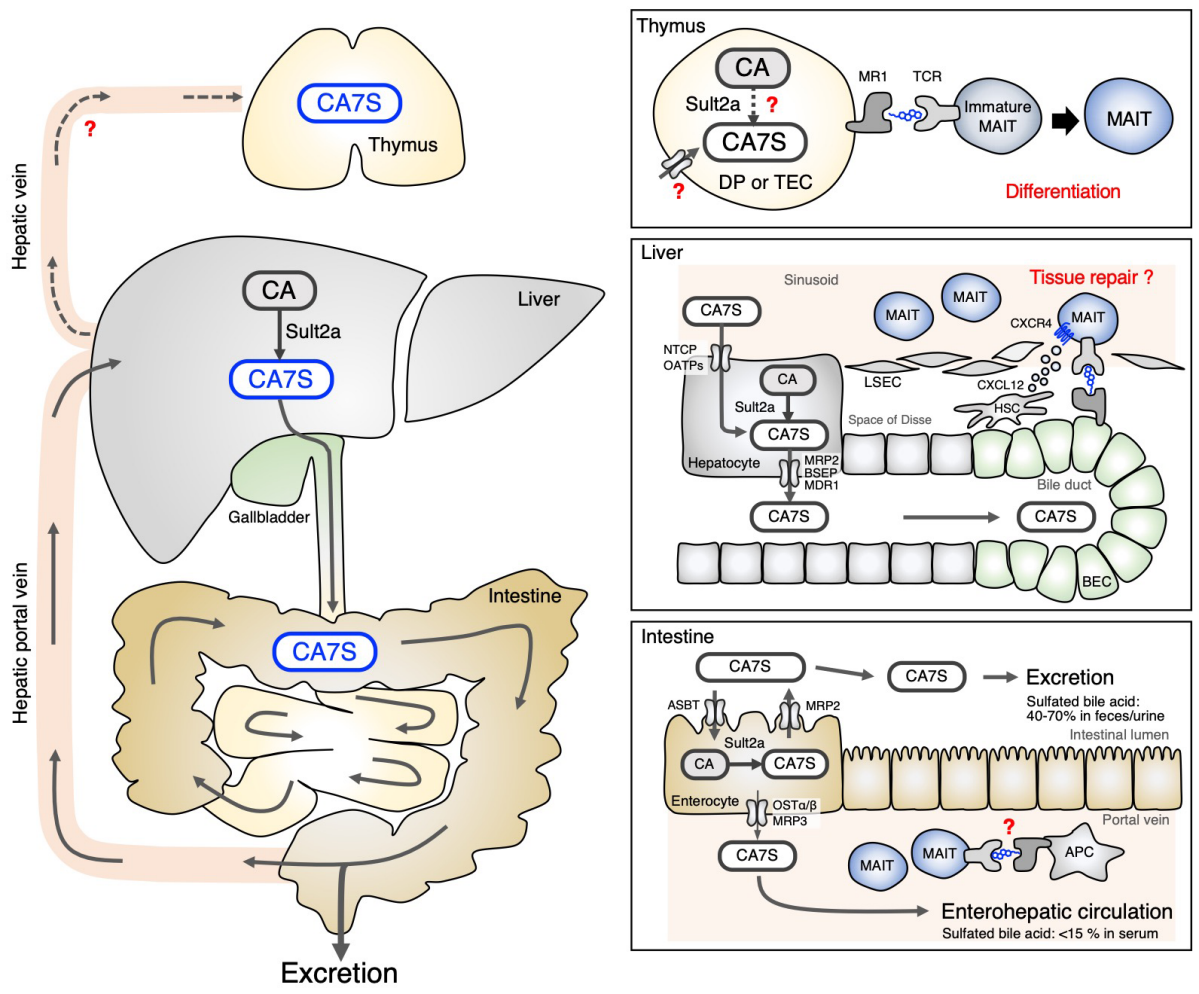
Circulation of CA7S in the body. CA7S is mainly biosynthesized in the liver from CA, stored in the gallbladder and excreted through the intestine (left). The source of thymic CA7S is unknown (Thymus). CA7S in the bile duct may be presented to liver MAIT cells surrounding biliary epithelial cells (BEC) expressing MR1 (Liver). Sult2a expressed by enterocytes can locally produce CA7S in some intestinal areas, which might control the homeostasis of intestinal MAIT cells. Enterocyte-derived CA7S may also be transported through the enterohepatic vein (Intestine). .

As CA7S is weakly recognized by MAIT cells, it may contribute to the development of MAIT cells in the thymus (**Figure 2**). CA7S was detected in the thymus, and in mice lacking all Sult2a isoforms, thymic development of MAIT cells was impaired (33). Among thymic MAIT cells, the most mature stage, stage 3 (CD44^+^CD24^−^) (10), was severely affected. In mature thymic MAIT cells, MAIT17 occurs more frequently than MAIT1, whereas MAIT17 was fewer than MAIT 1 in Sult2a-deficient mice (33). These phenotypes were similar to those of GF mice (8, 10). While CA7S has been detected in the thymus, it is still unclear whether CAS is taken up and presented by thymocytes (**Figure 2**). Alternatively, Sult2a is also expressed in thymus as well as hepatocytes, implying that CAS might be synthesized within thymocytes when its substrate CA is available (**Figure 2**). Conditional deletion of all Sult2a isoforms will answer the key question whether CAS is *de novo* generated in thymocytes.

### 3.4 Effect of CA7S on MAIT cells in peripheral tissues

The role of CA7S in MAIT cells in peripheral tissues is not well understood. Unlike GF mice, the number of MAIT cells in the liver was not significantly decreased in Sult2a-deficient mice (33), implying that the antigen(s) utilized for the maintenance of MAIT cells vary by tissue. However, among liver-resident T cells, only MAIT cells lost the expression of multiple T cell signature genes, which is in contrast to invariant natural killer T (iNKT) cells and conventional T cells. Thus, CA7S might play an important role in shaping the identity of MAIT cells in the liver. MAIT cell-dependent protective immunity in the absence of CA7S function would be a worthy subject of further investigation utilizing infection models. Liver MAIT cells localize in the hepatic sinusoid, close to bile duct where CA7S is abundantly present; however, it is currently unknown whether bile acid metabolites play a role in MAIT cells in ‘distant’ tissues, such as the skin or lungs. Quantitative analysis of bile acid metabolites in these tissues would be required for further clarification. Although Sult2a is mainly expressed in hepatocytes, its transcript is also highly detected in other tissues (42), such as in some regions of the small intestine (**Figure 2**) (42, 43). CA7S produced in non-liver tissues might contribute to the maintenance of MAIT cells locally, which is a potential area of further investigation.

In humans, instead of CA7S, CA3S is an abundant cholic acid sulfate (35). Although the position of sulfation is different, CA3S can be presented by MR1 and recognized by MAIT TCR. In contrast to 5-OP-RU which triggers proliferation of peripheral MAIT cells, CA3/7S only induces survival, not proliferation. Thus, CA3/7S appears to induce qualitatively distinct MAIT cell responses. Indeed, while 5-OP-RU upregulates pro-inflammatory genes, CA3/7S induces gene signatures characterized by homeostatic and tissue repair responses (33). Among these is *CXCR4*, which contributes to migration and residency of lymphocytes in the tissues. It is therefore possible that CA3/7S, which is abundant in bile, may contribute to the residency of MAIT cells in the liver sinusoid where MAIT cells are most enriched particularly in humans (16, 18, 19). Since the barrier composed of liver sinusoidal endothelial cells (LSEC) is fragile, upon liver damage, MAIT cells will likely come into contact with cholangiocytes/biliary epithelial cells (BEC) that express MR1 (16, 44). One might speculate that bile acid metabolites presented by MR1 on BEC promote tissue repair responses. Additionally, MAIT cells may act as a sensor of bile acid homeostasis.

Recently, roles of secondary bile acid metabolites, which are produced by the microbiota from primary bile acids, have been highlighted in T cell development. 3-oxo-litho cholic acid (3-oxo-LCA) has been shown to inhibit Th17 differentiation through the interaction with RORγt (45). Furthermore, the 3-oxo-LCA derivative, iso-allo-LCA, promotes regulatory T cell (Treg) differentiation via mitochondria-mediated epigenetic regulation (45) through Nr4a1 (46), which is supported by bacterial genetics (47). A similar secondary bile acid, iso-deoxycholic acid (iso-DCA), antagonizes FXR and the impairs immunogenic properties of dendritic cells, leading to pTreg maturation (48). In contrast to these indirect effects of secondary bile acids on T cells, CA3S and CA7S are endogenous primary bile acids that are directly recognized by TCR as antigens. Nevertheless, since CA3/7S also potentially act on nuclear receptor and/or GPCR families (39), sulfated bile acids may serve unknown pleiotropic functions within the immune system.

### 3.5 Role of CAS in disease settings

Thus far we have discussed CAS in a homeostatic context. However, quantitative variations in CAS have been reported in several diseases (35, 49–51). Furthermore, the expression of SULT2A1 is reported to be decreased in cholestatic diseases (52–55). Examination of the involvement of the CAS-MAIT cell axis in these disorders would be intriguing (18, 56). In particular, cholestatic autoimmune diseases, such as primary biliary cholangitis (PBC) and primary sclerosis cholangitis (PSC), have been associated with MAIT cells (57–60). The role of MAIT cells in immune diseases related to bile duct abnormalities is therefore an exciting area for future research.

## 4 Future perspective

Why should bile acids metabolites be recognized by MAIT cells? Currently, there is still no clear answer to this teleological question. The correlation of CAS-rich sites (bile duct) and MAIT cell-rich sites (liver sinusoid) raise several hypotheses regarding their role in tissue residency. As bile acids can sometimes harm the body, excessive cholic acids are continuously excreted as sulfated forms. Excreted forms are therefore stably and abundantly present in the body, which make them effective for the maintenance of a host cell lineage. Moreover, recycling ‘waste’ metabolites is considered as an efficient strategy to make use of limited metabolites in the body. Nevertheless, at present, the localization of MAIT cells in other tissues, such as lung and skin where CAS is presumably less abundant, cannot be simply explained by bile acids. Additionally, the discovery of the ‘peculiar’ antigenic structure of the bile acid skeleton may suggest that MR1 can present a far greater variety of molecules than previously assumed, and that the MAIT TCR, despite its lack of diversity, can recognize complexes of such diverse antigens with MR1. It is exciting to imagine that further diverse self-antigen(s) for MAIT cells are present in different tissues and regulate their tissue-specific adaptation, which warrants future studies.

## Author contributions

EI: Writing – original draft, Writing – review & editing. SY: Writing – original draft, Writing – review & editing.

## References

[1] Godfrey DI Uldrich AP Mccluskey J Rossjohn J Moody DB. The burgeoning family of unconventional T cells. Nat Immunol. (2015) 16:1114–23. doi: 10.1038/ni.3298 26482978

[2] Kjer-Nielsen L Patel O Corbett AJ Le Nours J Meehan B Liu L. MR1 presents microbial vitamin B metabolites to MAIT cells. Nature. (2012) 491:717–23. doi: 10.1038/nature11605 23051753

[3] Godfrey DI Koay HF McCluskey J Gherardin NA. The biology and functional importance of MAIT cells. Nat Immunol. (2019) 20:1110–28. doi: 10.1038/s41590-019-0444-8 31406380

[4] Legoux F Salou M Lantz O. MAIT cell development and functions: the microbial connection. Immunity. (2020) 53:710–23. doi: 10.1016/j.immuni.2020.09.009 33053329

[5] Treiner E Duban L Bahram S Radosavljevic M Wanner V Tilloy F. Selection of evolutionarily conserved mucosal-associated invariant T cells by MR1. Nature. (2003) 423:164–9. doi: 10.1038/nature01700 12634786

[6] Corbett AJ Eckle SBG Birkinshaw RW Liu L Patel O Mahony J. T-cell activation by transitory neo-antigens derived from distinct microbial pathways. Nature. (2014) 509:361–5. doi: 10.1038/nature13160 24695216

[7] Seach N Guerri L Le Bourhis L Mburu Y Cui Y Bessoles S. Double positive thymocytes select mucosal-associated invariant T cells. J Immunol. (2013) 191:6002–9. doi: 10.4049/jimmunol.1301212 24244014

[8] Legoux F Bellet D Daviaud C El Morr Y Darbois A Niort K. Microbial metabolites control the thymic development of mucosal-associated invariant T cells. Science. (2019) 366:494–9. doi: 10.1126/science.aaw2719 31467190

[9] Constantinides MG Link VM Tamoutounour S Wong AC Perez-Chaparro PJ Han SJ. MAIT cells are imprinted by the microbiota in early life and promote tissue repair. Science. (2019) 366:445. doi: 10.1126/science.aax6624 PMC7603427 31649166

[10] Koay HF Gherardin NA Enders A Loh L Mackay LK Almeida CF. A three-stage intrathymic development pathway for the mucosal-associated invariant T cell lineage. Nat Immunol. (2016) 17:1300–11. doi: 10.1038/ni.3565 27668799

[11] Bugaut H El Morr Y Mestdagh M Darbois A Paiva RA Salou M. A conserved transcriptional program for MAIT cells across mammalian evolution. J Exp Med. (2024) 221:e20231487. doi: 10.1084/jem.20231487 38117256 PMC10733631

[12] Hogquist KA Jameson SC Heath WR Howard JL Bevan MJ Carbone FR. T cell receptor antagonist peptides induce positive selection. Cell. (1994) 76:17–27. doi: 10.1016/0092-8674(94)90169-4 8287475

[13] Tanchot C Lemonnier FA Pérarnau B Freitas AA Rocha B. Differential requirements for survival and proliferation of CD8 naive or memory T cells. Science. (1997) 276:2057–62. doi: 10.1126/science.276.5321.2057 9197272

[14] Viret C Wong FS Janeway CA. Designing and maintaining the mature TCR repertoire. Immunity. (1999) 10:559–68. doi: 10.1016/s1074-7613(00)80055-2 10367901

[15] Pais Ferreira D Silva JG Wyss T Fuertes Marraco SA Scarpellino L Charmoy M. Central memory CD8+ T cells derive from stem-like Tcf7hi effector cells in the absence of cytotoxic differentiation. Immunity. (2020) 53:985–1000. doi: 10.1016/j.immuni.2020.09.005 33128876

[16] Jeffery HC Van Wilgenburg B Kurioka A Parekh K Stirling K Roberts S. Biliary epithelium and liver B cells exposed to bacteria activate intrahepatic MAIT cells through MR1. J Hepatol. (2016) 64:1118–27. doi: 10.1016/j.jhep.2015.12.017 PMC4822535 26743076

[17] Böttcher K Rombouts K Saffioti F Roccarina D Rosselli M Hall A. MAIT cells are chronically activated in patients with autoimmune liver disease and promote profibrogenic hepatic stellate cell activation. Hepatology. (2018) 68:172–86. doi: 10.1002/hep.29782 29328499

[18] Hegde P Weiss E Paradis V Wan J Mabire M Sukriti S. Mucosal-associated invariant T cells are a profibrogenic immune cell population in the liver. Nat Commun. (2018) 9:1–12. doi: 10.1038/s41467-018-04450-y 29858567 PMC5984626

[19] Lett MJ Mehta H Keogh A Jaeger T Jacquet M Powell K. Stimulatory MAIT cell antigens reach the circulation and are efficiently metabolised and presented by human liver cells. Gut. (2022) 71:2526–38. doi: 10.1136/gutjnl-2021-324478 PMC9664123 35058274

[20] Dusseaux M Martin E Serriari N Péguillet I Premel V Louis D. Human MAIT cells are xenobiotic-resistant, tissue-targeted, CD161 hi IL-17-secreting T cells. Blood. (2011) 117:1250–9. doi: 10.1182/blood-2010-08-303339 21084709

[21] Tang X-Z Jo J Tan AT Sandalova E Chia A Tan KC. IL-7 licenses activation of human liver intrasinusoidal mucosal-associated invariant T cells. J Immunol. (2013) 190:3142–52. doi: 10.4049/jimmunol.1203218 23447689

[22] Keller AN Eckle SBG Xu W Liu L Hughes VA Mak JYW. Drugs and drug-like molecules can modulate the function of mucosal-associated invariant T cells. Nat Immunol. (2017) 18:402–11. doi: 10.1038/ni.3679 28166217

[23] Harriff MJ McMurtrey C Froyd CA Jin H Cansler M Null M. MR1 displays the microbial metabolome driving selective MR1-restricted T cell receptor usage. Sci Immunol. (2018) 3:eaao2556. doi: 10.1126/sciimmunol.aao2556 30006464 PMC7085347

[24] Patel O Kjer-Nielsen L Le Nours J Eckle SBG Birkinshaw R Beddoe T. Recognition of vitamin B metabolites by mucosal-associated invariant T cells. Nat Commun. (2013) 4:1–9. doi: 10.1038/ncomms3142 23846752

[25] Eckle SBG Birkinshaw RW Kostenko L Corbett AJ McWilliam HEG Reantragoon R. A molecular basis underpinning the T cell receptor heterogeneity of mucosal-associated invariant T cells. J Exp Med. (2014) 211:1585–600. doi: 10.1084/jem.20140484 PMC4113946 25049336

[26] Salio M Awad W Veerapen N Gonzalez-Lopez C Kulicke C Waithe D. Ligand-dependent downregulation of MR1 cell surface expression. Proc Natl Acad Sci U. S. A. (2020) 117:10465–75. doi: 10.1073/pnas.2003136117 PMC7229755 32341160

[27] McWilliam HEG Villadangos JA. MR1 antigen presentation to MAIT cells and other MR1-restricted T cells. Nat Rev Immunol. (2023) 24:178–92. doi: 10.1038/s41577-023-00934-1 PMC11108705 37773272

[28] Young MH U’Ren L Huang S Mallevaey T Scott-Browne J Crawford F. MAIT cell recognition of MR1 on bacterially infected and uninfected Cells. PloS One. (2013) 8:6–11. doi: 10.1371/journal.pone.0053789 PMC3544856 23342002

[29] Crowther MD Dolton G Legut M Caillaud ME Lloyd A Attaf M. Genome-wide CRISPR–Cas9 screening reveals ubiquitous T cell cancer targeting via the monomorphic MHC class I-related protein MR1. Nat Immunol. (2020) 21:178–85. doi: 10.1038/s41590-019-0578-8 PMC6983325 31959982

[30] Lepore M Kalinichenko A Calogero S Kumar P Paleja B Schmaler M. Functionally diverse human T cells recognize non-microbial antigens presented by MR1. Elife. (2017) 6:1–22. doi: 10.7554/eLife.24476 PMC5459576 28518056

[31] Shibata K Motozono C Nagae M Shimizu T Ishikawa E Motooka D. Symbiotic bacteria-dependent expansion of MR1-reactive T cells causes autoimmunity in the absence of Bcl11b. Nat Commun. (2022) 13:1–5. doi: 10.1038/s41467-022-34802-8 36376329 PMC9663695

[32] Chancellor A Alan Simmons R Khanolkar RC Nosi V Beshirova A Berloffa G. Promiscuous recognition of MR1 drives self-reactive mucosal-associated invariant T cell responses. J Exp Med. (2023) 220:e20221939. doi: 10.1084/jem.20221939 37382893 PMC10309188

[33] Ito E Inuki S Izumi Y Takahashi M Dambayashi Y Ciacchi L. Sulfated bile acid is a host-derived ligand for MAIT cells. Sci Immunol. (2024) 9:eade6924. doi: 10.1126/sciimmunol.ade6924 38277465 PMC11147531

[34] Wang CJH Awad W Liu L Mak JYW Veerapen N Illing PT. Quantitative af fi nity measurement of small molecule ligand binding to major histocompatibility complex class-I – related. J Biol Chem. (2022) 298:1–12. doi: 10.1016/j.jbc.2022.102714 PMC9764189 36403855

[35] Alnouti Y. Bile acid sulfation: A pathway of bile acid elimination and detoxification. Toxicol Sci. (2009) 108:225–46. doi: 10.1093/toxsci/kfn268 19131563

[36] Shimohira T Kurogi K Liu MC Suiko M Sakakibara Y. The critical role of His48 in mouse cytosolic sulfotransferase SULT2A8 for the 7α-hydroxyl sulfation of bile acids. Biosci Biotechnol Biochem. (2018) 82:1359–65. doi: 10.1080/09168451.2018.1464897 29685090

[37] Teramoto T Nishio T Kurogi K Sakakibara Y Kakuta Y. The crystal structure of mouse SULT2A8 reveals the mechanism of 7α-hydroxyl, bile acid sulfation. Biochem Biophys Res Commun. (2021) 562:15–20. doi: 10.1016/j.bbrc.2021.04.113 34030040

[38] Dawson PA Karpen SJ. Intestinal transport and metabolism of bile acids. J Lipid Res. (2015) 56:1085–99. doi: 10.1194/jlr.R054114 PMC4442867 25210150

[39] Chaudhari SN Harris DA Aliakbarian H Luo JN Henke MT Subramaniam R. Bariatric surgery reveals a gut-restricted TGR5 agonist with anti-diabetic effects. Nat Chem Biol. (2021) 17:20–9. doi: 10.1038/s41589-020-0604-z PMC7891870 32747812

[40] Chaudhari SN Luo JN Harris DA Aliakbarian H Yao L Paik D. A microbial metabolite remodels the gut-liver axis following bariatric surgery. Cell Host Microbe. (2021) 29:408–24. doi: 10.1016/j.chom.2020.12.004 PMC7954942 33434516

[41] Cai J Sun L Gonzalez FJ. Gut microbiota-derived bile acids in intestinal immunity, inflammation, and tumorigenesis. Cell Host Microbe. (2022) 30:289–300. doi: 10.1016/j.chom.2022.02.004 35271802 PMC8923532

[42] Teubner W Meinl W Florian S Kretzschmar M Glatt H. Identification and localization of soluble sulfotransferases in the human gastrointestinal tract. Biochem J. (2007) 404:207–15. doi: 10.1042/BJ20061431 PMC1868804 17335415

[43] Alnouti Y Klaassen CD. Tissue distribution and ontogeny of sulfotransferase enzymes in mice. Toxicol Sci. (2006) 93:242–55. doi: 10.1093/toxsci/kfl050 16807285

[44] Banales JM Huebert RC Karlsen T Strazzabosco M LaRusso NF Gores GJ. Cholangiocyte pathobiology. Nat Rev Gastroenterol Hepatol. (2019) 16:269–81. doi: 10.1038/s41575-019-0125-y PMC6563606 30850822

[45] Hang S Paik D Yao L Kim E Jamma T Lu J. Bile acid metabolites control TH17 and Treg cell differentiation. Nature. (2019) 576:143–8. doi: 10.1038/s41586-019-1785-z PMC6949019 31776512

[46] Li W Hang S Fang Y Bae S Zhang Y Zhang M. A bacterial bile acid metabolite modulates Treg activity through the nuclear hormone receptor NR4A1. Cell Host Microbe. (2021) 29:1366–1377.e9. doi: 10.1016/j.chom.2021.07.013 34416161 PMC9064000

[47] Song X Sun X Oh SF Wu M Zhang Y Zheng W. Microbial bile acid metabolites modulate gut RORγ+ regulatory T cell homeostasis. Nature. (2020) 577:410–5. doi: 10.1038/s41586-019-1865-0 PMC7274525 31875848

[48] Campbell C McKenney PT Konstantinovsky D Isaeva OI Schizas M Verter J. Bacterial metabolism of bile acids promotes generation of peripheral regulatory T cells. Nature. (2020) 581:475–9. doi: 10.1038/s41586-020-2193-0 PMC7540721 32461639

[49] Shinohara T Muraji T Tsugawa C Nishijima E Satoh S Takamizawa S. Efficacy of urinary sulfated bile acids for diagnosis of bacterial cholangitis in biliary atresia. Pediatr Surg Int. (2005) 21:701–4. doi: 10.1007/s00383-005-1493-7 16096796

[50] Nanashima A Obatake M Sumida Y Abo T Yamane Y Nomura M. Clinical significance of measuring urinary sulfated bile acids in adult patients with hepatobiliary diseases. Hepato-Gastroenterology J Clin Res Pract. (2009) 56:299–302. 19579586

[51] Li W Alamoudi JA Gautam N Kumar D Olivera M Gwon Y. Urinary BA indices as prognostic biomarkers for complications associated with liver diseases. Int J Hepatol. (2022) 2022:5473752. doi: 10.1155/2022/5473752 35402050 PMC8986411

[52] Elekima OTL Mills CO Ahmad A Skinner GRB Ramsden DB Bown J. Reduced hepatic content of dehydroepiandrosterone sulphotransferase in chronic liver diseases. Liver. (2000) 20:45–50. doi: 10.1034/j.1600-0676.2000.020001045.x 10726960

[53] Yalcin EB More V Neira KL Lu ZJ Cherrington NJ Slitt AL. Downregulation of sulfotransferase expression and activity in diseased human livers. Drug Metab Dispos. (2013) 41:1642–50. doi: 10.1124/dmd.113.050930 PMC3876809 23775849

[54] Chai J Feng X Zhang L Chen S Cheng Y He X. Hepatic expression of detoxification enzymes is decreased in human obstructive cholestasis due to gallstone biliary obstruction. PloS One. (2015) 10:1–14. doi: 10.1371/journal.pone.0120055 PMC4370735 25798860

[55] Wunsch E Klak M Wasik U Milkiewicz M Blatkiewicz M Urasinska E. Liver expression of sulphotransferase 2A1 enzyme is impaired in patients with primary sclerosing cholangitis: lack of the response to enhanced expression of PXR. J Immunol Res. (2015) 2015:571353. doi: 10.1155/2015/571353 26504856 PMC4609469

[56] Jiang X Peng Y Liu L Wang Y Li M Li W. MAIT cells ameliorate liver fibrosis by enhancing the cytotoxicity of NK cells in cholestatic murine models. Liver Int. (2022) 42:2743–58. doi: 10.1111/liv.15445 36181707

[57] Jiang X Lian M Li Y Zhang W Wang Q Wei Y. The immunobiology of mucosal-associated invariant T cell (MAIT) function in primary biliary cholangitis: regulation by cholic acid-induced interleukin-7. J Autoimmun. (2018) 90:64–75. doi: 10.1016/j.jaut.2018.01.007 29429758

[58] Setsu T Yamagiwa S Tominaga K Kimura N Honda H Kamimura H. Persistent reduction of mucosal-associated invariant T cells in primary biliary cholangitis. J Gastroenterol Hepatol. (2018) 33:1286–94. doi: 10.1111/jgh.14076 29266628

[59] von Seth E Zimmer CL Reuterwall-Hansson M Barakat A Arnelo U Bergquist A. Primary sclerosing cholangitis leads to dysfunction and loss of MAIT cells. Eur J Immunol. (2018) 48:1997–2004. doi: 10.1002/eji.201847608 30252934

[60] ValestrandLZhengFHansenSHØgaardJHovJRBjörkströmNK. Bile from patients with primary sclerosing cholangitis contains mucosal-associated invariant T-cell antigens. Am J Pathol. (2022) 192:629–41. doi: 10.1016/j.ajpath.2021.12.008 35063408

